# Prevalence of associated injuries of spinal trauma and their effect on medical utilization among hospitalized adult subjects – a nationwide data-based study

**DOI:** 10.1186/1472-6963-9-137

**Published:** 2009-08-03

**Authors:** Dachen Chu, Yi-Hui Lee, Ching-Heng Lin, Pesus Chou, Nan-Ping Yang

**Affiliations:** 1 Community Medicine Research Center & Department and Institute of Public Health, National Yang-Ming University, Taipei, Taiwan, Republic of China; 2 Department of Neurosurgery, Taipei City Hospital, Taipei, Taiwan, Republic of China; 3 The School of Nursing, Chang-Gung University, Tao-Yuan, Taiwan, Republic of China; 4 Department of Geriatrics & Department of Orthopedic Surgery, Tao-Yuan General Hospital, Department of Health, Tao-Yuan, Taiwan, Republic of China

## Abstract

**Background:**

This study was wanted to investigate the prevalence of concomitant injuries among hospitalized acute spinal trauma patients aged 20 and over and the effects of those injuries on medical utilization in Taiwan.

**Methods:**

Nationwide inpatient datasets of Taiwan's National Health Insurance (NHI) database from between 2000 and 2003 were used. The major inclusion criteria used to select cases admitted due to acute spinal trauma were based on three diagnostic International Classification of Disease, 9^th ^Version (ICD-9) codes items: (1) fracture of vertebral column without mention of spinal cord injury; (2) fracture of vertebral column with spinal cord injury; or (3) spinal cord lesion without evidence of spinal bone injury. To investigate the associated injuries among the eligible subjects, the concomitant ICD-9 diagnosis codes were evaluated and classified into six co-injury categories: (1) head trauma; (2) chest trauma; (3) abdominal trauma; (4) pelvic trauma; (5) upper extremities trauma; (6) lower extremities trauma.

**Results:**

There were 51,641 cases studied; 27.6% of these subjects suffered from neurological deficit, but only 17.3% underwent a surgical procedure for spinal injury. Among them, the prevalence of associated injuries were as follows: head trauma, 17.2%; chest injury, 2.9%; abdominal trauma, 1.5%; pelvic injury or fracture, 2.5%; upper limb fracture, 4.4%; lower limb fracture, 5.9%. The three major locations of acute spinal injury (cervical, thoracic, or lumbar spine) were found to be combined with unequal distributions of associated injuries. By stepwise multiple linear regression, gender, age, location of spinal injury, neurological deficit, surgical intervention and the six combined injuries were identified significantly as associated factors of the two kinds of medical utilization, length of stay (LOS) and direct medical cost. The combinations of acute spinal trauma with lower extremity injury, pelvic injury, chest injury, abdominal injury and upper extremity injury resulted in of the highest utilization of medical resources, the estimated additional LOS being between 4.3 and 1.2 days, and the extra medical cost calculated as being between $1,230 and $320.

**Conclusion:**

The occurrence of associated Injuries among hospitalized acute spinal trauma patients in Taiwan is not uncommon, and results in an obvious effect on medical utilization.

## Background

Vertebral injuries are usually a result of trauma, and some spinal injury patients also sustain injury to other parts of the body in addition to spinal trauma. Patients with a traumatic spine injury and polytrauma have poorer short- and long-term outcomes [1]. Traumatic spine injuries with polytrauma always involve multiple injuries of varying severity that influence medical utilization, but the incidence of these associated injuries is rarely discussed. Based on the nationwide insurance database, diagnoses of injury and poisoning have been found to be the most common diagnostic categories (26.4% of all cases) for patients of emergency units in Taiwan [2], and the average incidence of acute spinal trauma in Taiwan has been calculated to be 61.61/100,000, and is similar in both genders (rate ratio of male to female: 0.99) [3]. However, a descriptive and analytic study of the associated injuries of these spinal trauma subjects has not been made. Therefore, the aim of this study was to investigate the prevalence of concomitant injuries among hospitalized acute spinal trauma patients aged 20 and over and the effects of those injuries on medical utilization in Taiwan.

## Methods

### The targeted study-population

In Taiwan, the National Health Insurance (NHI) Plan has accumulated 23.75 million administrative and claims data, the largest dataset of its kind in the world. The National Health Research Institute (NHRI), in cooperation with the National Health Insurance Bureau (NHIB), has established a NHI research database (NHIRD). Specific datasets of the NHI research database, including 'monthly claims summary for inpatient claims,' 'inpatient cost by admissions,' and 'details of inpatient orders,' from between 2000 and 2003 were analyzed. Three exclusive criteria and three inclusive criteria were used to select those cases admitted due to acute spinal trauma [3]. The exclusive criteria were: (1) chronic length of stay (LOS) ≥ 1 and acute LOS = 0; (2) the specialist, the physician responsible for the studied case, other than surgery, orthopedics, neurosurgery, neurology and emergency; and (3) cases with a discharge status coded as reference, against-advice discharge (AAD) or escape. In addition, those who were admitted for subsequent surgical procedures, such as removal of spinal implanted devices or debridement, were also excluded. The inclusive criteria were the following three International Classification of Disease, 9^th ^Version (ICD-9) code items: (1) 805.X, i.e., 805.00–805.9 (fracture of vertebral column without mention of spinal cord injury); (2) 806.X, i.e., 806.00–806.9 (fracture of vertebral column with spinal cord injury); and (3) 952.X, i.e., 952.00–952.9 (spinal cord lesion without evidence of spinal bone injury).

### Data protection and permission

Data in the NHIRD that could be used to identify patients or care providers, including medical institutions and physicians, is scrambled before being sent to the NHRI for database construction and is further scrambled before being released to each researcher. Theoretically, it is impossible to query the data alone to identify individuals at any level using this database. All researchers who wish to use the NHIRD and its data subsets are required to sign a written agreement declaring that they have no intention of attempting to obtain information that could potentially violate the privacy of patients or care providers. This study had been evaluated and agreed by the NHRI to analyze their NHIRD (Application and Agreement Number: 94120). Otherwise, this study was premised and supported by the grants from the Taoyuan General Hospital, Department of Health, Taiwan (DOH-PTH-9729).

### Definition of associated injuries

In order to investigate the associated injuries of the hospitalized acute spinal trauma subjects included in this study, the concomitant ICD-9 diagnosis codes were evaluated and classified into six co-injury categories. The first co-injury, head trauma, was defined as coded 800–804 (fracture of skull) or 850–854 (intracranial injury, excluding those with skull fracture). The second co-injury, chest trauma, was defined as coded 807 (fracture of ribs, sternum, larynx and trachea), 860 (traumatic pneumothorax and hemothorax), 861 (injury to heart and lung), or 862 (injury to other and unspecified intra-thoracic organs). The third, abdominal trauma, was defined as coded 863 (injury to gastrointestinal tract), 864 (injury to liver), 865 (injury to spleen), 866 (injury to kidney), or 868 (injury to other intra-abdominal organs). The fourth, pelvic trauma, was defined as coded 808 (fracture of pelvis) or 867 (injury to pelvic organs). The fifth and sixth co-injuries, extremities trauma, were defined as coded 810–819 (fracture of upper limb) and 820–829 (fracture of lower limb), respectively.

### Safety and quality control of data regarding medical services

In Taiwan's NHI system, the NHRI safeguards the privacy and confidentiality of subjects whose data is used in research, and the NHIB has established a uniform system to control the quality of medical services and coding. If the medical services provided by the contracted medical care institution to the beneficiaries were determined by the Professional Peer Review Committee to be incompatible with the provisions of the NHI Act, the expenses thereof are borne by the contracted medical care institutions themselves. In cases where drugs, laboratory tests or diagnostic examinations are provided by other contracted medical care institutions in accordance with the physician's instruction, and the insurer, after examination according to the rules of examination as described in the preceding article, decides not to pay the cost due to the physician's improper instruction, such expenses incurred thereof shall be borne by the medical institution where the physician practices. Otherwise, the Disputes Settlement Board, established under the National Health Insurance scheme, settles disputes arising from cases approved by the insurer and raised by the insured, group insurance applicants or contracted medical care institutions.

### Statistics

Descriptive statistics are presented as number of cases, percentage and mean with standard deviation (SD). The independent *t*-test and Pearson's chi-square test were used to evaluate the significance of differences; the odds ratio (OR) and its 95% confidence interval (CI) were calculated in order to compare the differences between genders, and the Mantel-Haenszel (M-H) χ^2 ^test was employed to examine trends. By analysis using stepwise multiple linear regression, the factors associated with medical utilization were classified. All of the statistical calculations were performed using the Statistical Package for Social Sciences for Windows (SPSS for Windows 13.0).

## Results

**Figure 1:**
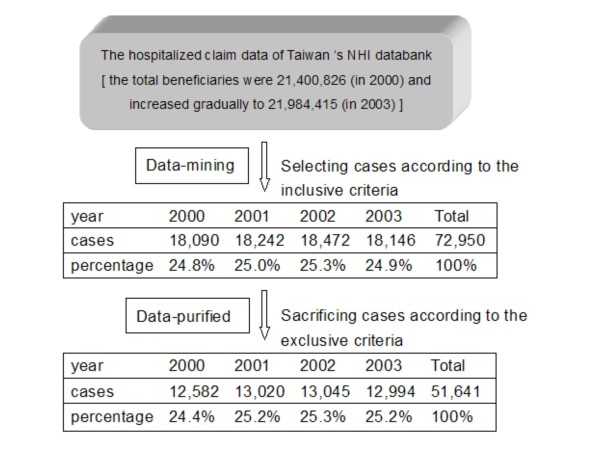
**The stepwise cases identification from the nationwide dataset among 2000–2003**.

Based on the above inclusion criteria, total 72,950 subjects aged 20 years and over were initially selected from the hospitalized claim data of Taiwan's NHI databank between 2000 and 2003. Through screening using the exclusion criteria, the present study totaled 51,641 cases (12,582 in 2000, 13,020 in 2001, 13,045 in 2002, and 12,994 in 2003) as the targeted population which represented all the patients, aged 20 years and over, who were admitted into the hospitals in Taiwan due to acute spinal trauma (figure 1).

Table 1 shows the characteristics of the 51,641 subjects included in the present study between 2000 and 2003. In general, these hospitalized acute spinal trauma subjects stayed in hospital for 8.5 ± 8.8 days and incurred a cost of $NT42,829.4 ± 79,394.1 (equal to $US1,360 ± 2,520). Nearly half of the studied cases (48.7%) were aged 60 years and over. The most common spinal trauma location among the patients included was the lumbar spine (57.0%), followed by the thoracic spine (22.9%). The ICD-9 code items 806.X or 952.X are defined as indicating neurological deficit; therefore, 27.6% of all the enrolled patients were cases of spinal trauma with neurological injury. In addition, the codes for any surgical intervention administered were checked in all the studied cases, and were found to include: (1) decompression, as ICD-9 procedure code 03.X (i.e., 03.0, 03.09, 03.53 or 03.59) or 79.29; (2) fusion, as 81.0× (i.e., 81.00–81.09); (3) instrumentation, as 78.59, 79.19 or 79.39; (4) bone graft, as 78.09 or 78.49; (5) disc management, as 80.5× (i.e., 80.50–80.59); and (6) C-spine traction, as 02.94; overall, 17.3% of the enrolled subjects underwent at least one of these surgical procedures. The above-mentioned characteristics, including age distribution, diagnostic categories, location of spinal lesion, incidence of spinal surgery and the consequent medical utilization differed significantly between genders.

**Table 1:** Characteristics of adult hospitalized acute spinal trauma patients in Taiwan, 2000–2003.

	Male		Female			Total	
				
	No., Mean	(%), S.D.	No., Mean	(%), S.D.	p value	No., Mean	(%), S.D.
General							
Enrolled subjects	25071	(48.5)	26579	(51.5)		51641	(100.0)
Length of stay (days)	9.2	9.8	7.8	7.6	<0.001	8.5	8.8
Medical cost ($NT)	51249.1	93168.0	34884.7	62696.8	<0.001	42829.4	79394.1
Age (years)							
20–39	7740	(64.9) ^a^	4194	(35.1)	<0.001	11934	(23.1)
40–59	8036	(55.2)^a^	6532	(44.8)		14568	(28.2)
≥ 60	9295	(37.0)^a^	15844	(63.0)		25139	(48.7)
ICD-9 codes ^d^							
805.X	16434	(65.5) ^b^	20985	(79.0)^b^	<0.001	37419	(72.5)
806.X	3374	(13.5)	3065	(11.5)		6439	(12.5)
952.X	5263	(21.0)	2520	(9.5)		7783	(15.1)
Location of lesion							
Cervical spine	6791	(27.8)^b^	2860	(10.8)^b^	<0.001	9831	(19.0)^c^
Thoracic spine	4316	(17.2)	7508	(28.3)	<0.001	11824	(22.9)^c^
Lumbar spine	13482	(53.8)	15972	(60.1)	<0.001	29454	(57.0)^c^
Sacral spine	860	(3.4)	2164	(8.1)	<0.001	3024	(5.9)^c^
Spinal surgery performed							
Yes	4809	(19.2) ^b^	4115	(15.5)^b^	<0.001	8924	(17.3)
No	20262	(80.8)	22455	(84.5)		42717	(82.7)

S.D.: standard deviation.

a: percentile within the same age stratum.

b: percentile within the same gender.

c: there were 1471 (2.8%) cases of incomplete injury location information due to incomplete coding, and 3901 (7.6%) cases of multiple-location injury were found.

d: 805.X, i.e., 805.00–805.9 (fracture of vertebral column without mention of spinal cord injury); 806.X, i.e., 806.00–806.9 (fracture of vertebral column with spinal cord injury); and 952.X, i.e., 952.00 – 952.9 (spinal cord lesion without evidence of spinal bone injury).

Among those enrolled in the study, 28.8% had extra-spinal injuries, and the prevalence of the associated injuries were as follows: head trauma, 17.2%; chest injury, 2.9%; abdominal trauma, 1.5%; pelvic injury or fracture, 2.5%; upper limb fracture, 4.4%; and lower limb fracture, 5.9%. There seemed to be a trend of decreasing occurrence of concomitant injury of central body locations (Table 2). Associated injuries were in general much more prevalent in males than in females who had suffered spinal trauma, the male-to-female odds ratios (OR) comparing the prevalence of associated head trauma, chest trauma, abdominal trauma, upper extremity trauma and lower extremity trauma being 1.71, 2.12, 2.02, 1.46 and 1.60, respectively; the only exception was pelvic trauma, for which the prevalence was higher in females than in males (equivalent OR = 1.18) (Table 2).

**Table 2:** The associated injuries of adult hospitalized acute spinal trauma patients in Taiwan, 2000–2003 (valid n = 51,641).

Type of associated injury		No.	%	O.R. (male *vs. *female)	(95% C.I.)	General prevalence
Head	male	5308	21.2	1.71	(1.64~1.80)	17.2%
	female	3599	13.5			
Chest	male	982	3.9	2.12	(1.90~2.37)	2.9%
	female	501	1.9			
Abdomen	male	494	2.0	2.02	(1.74~2.35)	1.5%
	female	262	1.0			
Pelvis	male	575	2.3	0.85	(0.76~0.95)	2.5%
	female	715	2.7			
Extremities, upper	male	1306	5.2	1.46	(1.34~1.59)	4.4%
	female	964	3.6			
Extremities, lower	male	1808	7.2	1.60	(1.48~1.72)	5.9%
	female	1232	4.6			

O.R.: odds ratio

C.I.: confidence interval

The different locations of the acute spinal injury (cervical, thoracic, lumbar or sacral spine) were found to be combined with unequal distributions of associated injuries, as shown in Table 3. In particular, patients with spinal injuries at the C-spine level sustained a noticeably higher percentage of associated head trauma (42% in general). Chest trauma was more commonly found in combination with C-spine and T-spine injuries, upper extremities trauma with C-spine injury, and both pelvic trauma and lower extremities trauma with L-spine injury. Otherwise, the cases of spinal injury at the T- or L-spine level were associated with a lower prevalence of the six types of associated injures with age stratum (p < 0.001 in the trend test).

**Table 3:** Prevalence (%) of associated injuries among the three major spinal trauma locations in different age strata.

	Head Trauma	Chest Trauma	Abdomen Trauma	Pelvis Trauma	Upper Extremities Trauma	Lower Extremities Trauma	p value for χ^2^test
C-spine							
In general	42.5	3.7	1.2	1.0	5.1	4.8	<0.001
20–39 y/o	49.0	2.8	1.7	1.2	5.5	4.7	
40–59 y/o	40.4	4.2	1.2	1.1	5.1	5.2	
60 y/o and over	36.2	4.5	0.4	0.7	4.4	4.4	
p value for M-H χ^2 ^test	<0.001	<0.001	<0.001	0.11	0.06	0.70	
T-spine							
In general	10.2	3.7	1.1	1.7	4.2	4.5	<0.001
20–39 y/o	17.3	7.1	3.1	3.2	7.0	8.5	
40–59 y/o	15.2	6.4	1.7	2.0	6.2	5.3	
60 y/o and over	7.1	2.1	0.5	1.3	3.0	3.5	
p value for M-H χ^2 ^test	<0.001	<0.001	<0.001	<0.001	<0.001	<0.001	
L-spine							
In general	9.7	2.4	1.5	2.6	4.1	6.7	<0.001
20–39 y/o	14.9	4.3	4.6	5.3	6.8	13.4	
40–59 y/o	12.6	3.3	1.8	3.1	4.7	8.8	
60 y/o and over	6.8	1.3	0.4	1.5	3.0	3.7	
p value for M-H χ^2 ^test	<0.001	<0.001	<0.001	<0.001	<0.001	<0.001	

The effects of the associated injuries of acute spinal trauma patients on consequent medical utilization were evaluated (Table 4). By stepwise multiple linear regression analysis, gender, age, location of spinal injury, neurological deficit (defined as ICD-9 codes recorded as 806.X or 952.X), surgical intervention and the six combined injuries were found to be factors that have a significant impact on the two types of medical utilization, LOS and direct medical cost. Administration of spinal surgical intervention was the most important factor in increasing the medical cost and LOS. Among the six associated injury categories, the combinations of spinal trauma with lower extremity injury, pelvic injury, chest injury, abdominal injury and upper extremity injury resulted in the greatest use of medical resources, the estimated additional LOS being 4.3, 3.6, 3.5, 2.4 and 1.2 days, respectively, and the extra medical cost calculated as being between $1,230 and $320. Perhaps the effect of an associated head injury could be corrected by other associated factors, such as higher percentage of C-spine location. Higher spinal trauma locations, i.e., C-spine and T-spine rather than L-spine injury, require a significantly greater amount of medical care.

**Table 4:** The length of stay, direct medical cost and related factors of adult hospitalized acute spinal trauma patients in Taiwan, 2000–2003.

	length of stay (days)			medical cost ($NT)		
		
Dependant variables\Independent variables	β	(s.e.)	p value	β	(s.e.)	p value
Multiple linear regression model:						
Sex (M/F)	0.67	0.08	<0.001	5891.1	655.7	<0.001
Age (years)	0.03	0.002	<0.001	103.1	17.9	<0.001
Location of spinal injury						
C-spine (*vs. *L-spine)	3.23	0.13	<0.001	25984.5	1016.2	<0.001
T-spine (*vs. *L-spine)	0.70	0.09	<0.001	3821.5	751.8	<0.001
Neurological deficit (Y/N)^a^	0.85	0.11	<0.001	6515.1	853.9	<0.001
Spine surgical intervention (Y/N)	7.29	0.10	<0.001	115424.0	806.2	<0.001
Combined associated injuries						
head injury (Y/N)	-0.73	0.11	<0.001	-2410.2	901.5	0.008
chest injury (Y/N)	3.51	0.23	<0.001	29514.3	1853.1	<0.001
abdominal injury (Y/N)	2.40	0.33	<0.001	23025.6	2665.8	<0.001
pelvic injury (Y/N)	3.57	0.27	<0.001	15314.4	2157.6	<0.001
upper extremity injury (Y/N)	1.19	0.19	<0.001	10211.7	1506.7	<0.001
lower extremity injury (Y/N)	4.30	0.16	<0.001	38770.3	1309.5	<0.001
Constant	4.05			6308.6		

s.e.: standard error

n.s.: not significant

Note: the conversion rate used was approximately $US 1.0 being equivalent to $NT 30.0–33.0.

a: positive neurological deficit was defined as ICD-9 codes 806.X or 952.X.

## Discussion

The present study is aimed to define and identify all patients who have acute spinal injures within a complete population. Divided by the annual beneficiaries aged 20 years and over [4], the authors of the present study calculate the treated incidence of acute hospitalized spinal trauma of the adult population in Taiwan that were 83.38/100,000, 84.32/100,000, 82.59/100,000 and 80.87/100,000 in 2000, 2001, 2002 and 2003, respectively. There was a similar cross-section observational study of incident spinal fractures using an administrative data-base (the Manitoba Health Services Insurance Plan, MHSIP in Canada) and identifying cases coded as 805.X and 806.X during 1981–1984, that revealed an annual incidence rate of 64 per 100,000 [5]. Compared to the above study, the present epidemiological study in Taiwan still presented a higher incidence rate of spinal trauma even adjusted to the same definition (85% of the total studied cases were coded as 805.X and 806.X in the present study).

The Abbreviated Injury Scale (AIS) and the Injury Severity Score (ISS) are important tools for grading the severity of injury to trauma patients. An AIS approach is based on six anatomic regions of injury, including head/neck, face, thorax, abdomen, extremity and external soft tissue [6]. ISS, determined by rating each injury with the AIS, was proved to correlate substantially with mortality for the most severe injured cases [7,8]. Modified from the ISS and AIS approach to the anatomic categorizations, the present study classified those associated injures into six domains that consisted of head, thorax, abdomen, pelvis, upper and lower extremities.

In the US, a consecutive sample of 5,711 subjects entered onto the National Spinal Cord Injury (SCI) Database between 1986 and 1995 was analyzed in order to estimate the incidence of associated extraspinal fractures, and it was found that, of these subjects, 1,585 (28%) had sustained extraspinal fractures [9]. A study from the opposing viewpoint showed similar results: retrospective chart analysis of 590 multiple-trauma patients within a 4-year period revealed that 31% (n = 183) of the subjects also had a spine injury [10]. Another longitudinal study of the data of 508 consecutive hospital admissions due to spine trauma identified the presence of associated injuries in 240 (47%) cases, most frequently involving head (26%), chest (24%), or long bone injury (23%) [11]. In the present study, 28.8% of all the hospitalized acute spinal injury Taiwanese subjects studied had sustained extraspinal injury, and 17.4% of these had suffered more than one associated injury; these results show a similar prevalence of associated injury in spinal trauma patients to those reported previously, but also possibly a higher percentile of multiple associated injuries. A 6-year retrospective study carried out in the Level-I Trauma and Regional Spinal Cord Injury Center, New Jersey, USA, revealed that, of 1,672 patients who had been hit by a motor vehicle, 135 (8%) were found to have sustained spinal injury, 35% cervical injury, 19% thoracic injury, 37% lumbar injury, and 27% sacral injury [12]. On the contrary, over half of the spinal injuries in the cases included in the current study were found to have been injuries to the lumbar area, and fewer patients with cervical spine injuries were admitted during the studied period.

Spinal injury, in particular cervical spine injury, has been reported as being commonly found in combination with head injury. Associated head trauma was investigated in 188 patients with cervical spine and/or spinal cord injuries, and 35% were found to have moderate or severe head injuries [13]. A study of 359 admissions for C-spine injury (CSI) and head injury (HI) reported the coincidence of "primary" CSI with HI to be 24% [14]. The present study of Taiwanese subjects revealed the highest percentile for acute C-spinal injury with associated head injury. Another retrospective review of 1290 patients presenting with an acute vertebral fracture and spinal cord or cauda equina injury reported that, overall, 128 (10%) patients sustained 203 associated skeletal fractures [15]. The prevalence of associated extremity fractures (including upper and lower limbs) in the acute spinal trauma cases examined in the present study was found to be 10.3%, a result similar to that of the aforementioned study. In contrast, however, a lower prevalence of associated blunt abdominal trauma (or even a combination of abdominal trauma and pelvic trauma, 4,0%) was found as compared with previous studies: for example, a trauma registry and record review was performed between January 1998 and December 2005, which reported that intra-abdominal and hollow viscus injuries were found in 15% and 6%, respectively, of 292 patients with blunt spinal cord injury (SCI) [16]. Another retrospective review of 258 blunt trauma patients with lumbar spine fractures showed that only in 10.1% of cases were concomitant lumbar spine fractures and abdominal injuries sustained [17]. Within an available complete dataset of 275 acetabulum fracture patients, the incidence of concomitant acetabulum and spine fractures was identified as approximately 13% [18]; however, analyzed from the opposite direction, the present study showed that only 2.5% of the hospitalized spinal injury patients in Taiwan also sustained pelvic trauma.

Retrospective data analysis of 1,121 motorcyclists and 2,718 car occupants involved in automotive trauma showed that spinal injury occurred in 126 (11.2%) motorcyclists and 383 (14.1%) car occupants, the median hospital stay being 11.5 days (range of 0–235 days) and 10 days (range of 0–252 days), respectively [19,20]. A shorter mean admission duration was found in our study. The cost of treatment of acute spinal injuries is largely unknown and has rarely been studied. However, a US study based on the Nationwide Inpatient Sample (NIS) database revealed that there were 53,066 hospital admissions for osteoporotic vertebral fractures in women in 1997, the mean cost of each admission being $US 9,532 [21]. Another prospective study was performed between 1994 and 1998 in Minnesota, USA, and included forty-seven consecutive patients with a stable thoracolumbar burst fracture and no neurological deficit, who were randomized into one of two treatment groups: operative or non-operative treatment. The average charge per case for those managed operatively and non-operatively was reported as $49,063 (ranging from $26,517 to $102,583) and $11,264 (ranging from $4,686 to $20,891), respectively [22]. In comparison with the results of the above two studies, the mean cost of managing hospitalized acute spinal injured cases in Taiwan has been found to be much lower, at only 10–15% of the cost reported in the US. As for Europe, a survey of 200 patients hospitalized between 1997 and 2002 with traumatic thoracolumbar spine fractures was performed in The Netherlands. This study showed that stable fractures without neurological deficit were generally treated non-operatively, at a cost of EUR 5,100; unstable fractures without neurological deficit were treated either non-operatively, with an average of 29 hospitalization days and at an average cost of EUR 12,500, or operatively, with 24 hospitalization days and at an average cost of EUR 19,700; and unstable fractures with neurological deficit were usually treated operatively, at an average cost of EUR 31,900 [23]. Comparing the medical costs reported in the present study and those of the study performed in The Netherlands, using our linear regression model to assume a similar situation, only 4–14% of the cost in The Netherlands was expended in Taiwan.

This study had some limitations. First, the study used a dataset from the NHI database in Taiwan; for all these cases, the medical records (including final diagnoses, treatments, medications, etc.) were completed by medical personnel and the fixed payments for all procedures were applied by the medical facilities. The accuracy of diagnosis and the quality of the medical services is monitored by the NHIB, but no exact evaluations were available. Second, the value of the Taiwanese currency fluctuated between 2000 and 2003. In order to make a fair comparison, a single, acceptable mean for the conversion rate could be used.

## Conclusion

The occurrence of associated injuries among hospitalized acute spinal trauma patients in Taiwan is not uncommon, and the prevalence of associated injuries were as follows: head trauma, 17.2%; chest injury, 2.9%; abdominal trauma, 1.5%; pelvic injury or fracture, 2.5%; upper limb fracture, 4.4%; lower limb fracture, 5.9%. The presence of associated injuries had an obvious influence on the two major types of medical utilization, LOS and direct cost, and among the six associated injury categories, the combinations of spinal trauma with lower extremity injury, pelvic injury, chest injury, abdominal injury and upper extremity injury resulted in the highest utilization of medical resources, the additional LOS being estimated as 4.3, 3.6, 3.5, 2.4 and 1.2 days, respectively, and the extra medical cost calculated as being between $1,230 and $320.

## Competing interests

The authors declare that they have no competing interests.

## Authors' contributions

This manuscript represents original work, and the following authors designed the study (DC, NPY), gathered and analyzed the data (YHL, CHL), wrote the initial draft (DC, NPY), and ensured the accuracy of the data and analysis (PC, NPY). All the authors participated in the preparation of the manuscript and approved the final version.

## Pre-publication history

The pre-publication history for this paper can be accessed here:


